# Isolation and Characterization of Highly Pure Type A Spermatogonia From Sterlet (*Acipenser ruthenus*) Using Flow-Cytometric Cell Sorting

**DOI:** 10.3389/fcell.2021.772625

**Published:** 2021-12-10

**Authors:** Xuan Xie, Tomáš Tichopád, Galina Kislik, Lucie Langerová, Pavel Abaffy, Radek Šindelka, Roman Franěk, Michaela Fučíková, Christoph Steinbach, Mujahid Ali Shah, Ivo Šauman, Fan Chen, Martin Pšenička

**Affiliations:** ^1^ South Bohemian Research Center of Aquaculture and Biodiversity of Hydrocenoses, Faculty of Fisheries and Protection of Waters, Research Institute of Fish Culture and Hydrobiology, University of South Bohemia in České Budějovice, Vodňany, Czechia; ^2^ Imaging Methods Core Facility at BIOCEV, Operated by Faculty of Science, Charles University in Prague, Vestec, Czechia; ^3^ Laboratory of Gene Expression, Institute of Biotechnology of the Czech Academy of Sciences, Vestec, Czechia; ^4^ Biology Center of the Czech Academy of Sciences, Institute of Entomology, České Budějovice, Czechia; ^5^ University of South Bohemia, Faculty of Science, České Budějovice, Czechia; ^6^ Department of Pharmacology, C_DAT, University Medicine Greifswald, Greifswald, Germany

**Keywords:** fluorescence-activated cell sorting, spermatogonia, sturgeon, PLZF, germ stem cell, gonad

## Abstract

Sturgeons are among the most ancient linages of actinopterygians. At present, many sturgeon species are critically endangered. Surrogate production could be used as an affordable and a time-efficient method for endangered sturgeons. Our study established a method for identifying and isolating type A spermatogonia from different developmental stages of testes using flow cytometric cell sorting (FCM). Flow cytometric analysis of a whole testicular cell suspension showed several well-distinguished cell populations formed according to different values of light scatter parameters. FCM of these different cell populations was performed directly on glass slides for further immunocytochemistry to identify germ cells. Results showed that the cell population in gate P1 on a flow cytometry plot (with high forward scatter and high side scatter parameter values) contains the highest amount of type A spermatogonia. The sorted cell populations were characterized by expression profiles of 10 germ cell specific genes. The result confirmed that setting up for the P1 gate could precisely sort type A spermatogonia in all tested testicular developmental stages. The P2 gate, which was with lower forward scatter and side scatter values mostly, contained type B spermatogonia at a later maturing stage. Moreover, expressions of *plzf, dnd*, *boule,* and *kitr* were significantly higher in type A spermatogonia than in later developed germ cells. In addition, *plzf* was firstly found as a reliable marker to identify type A spermatogonia, which filled the gap of identification of spermatogonial stem cells in sterlet. It is expected to increase the efficiency of germ stem cell culture and transplantation with *plzf* identification. Our study thus first addressed a phenotypic characterization of a pure type A spermatogonia population in sterlet. FCM strategy can improve the production of sturgeons with surrogate broodstock and further the analysis of the cellular and molecular mechanisms of sturgeon germ cell development.

## Introduction

Sturgeons belong to the order Acipenseriformes, which are among the most ancient actinopterygians (Bemis et al., 1997). Sturgeons are called “living fossils,” and can be traced back to the Upper Cretaceous, diverging from an ancient, pre-Jurassic teleost lineages approximately 300 million years ago (Inoue et al., 2005). Since the late 1980s natural stocks of sturgeon species have decreased markedly. Most species of the Acipenseriformes are listed as threatened, mostly endangered or critically endangered, in the International Union for Conservation of Nature and Natural Resources Red List (IUCN 2020), due to habitat alteration caused by damming of rivers, pollution and overharvesting (Bronzi and Rosenthal, 2014). Although controlled propagation has been conducted successfully and has proved to be important for conservation, most species of sturgeon are late maturing, making culture and conservation costly and time consuming (Bemis and Kynard, 1997; Zhuang et al., 1997). Thus, new methods are needed to promote artificial propagation of sturgeons.

In past decades, germ cell xenotransplantation has been successfully established in rainbow trout (*Oncorhynchus mykiss*) and masu salmon (*Oncorhynchus masou*) (Takeuchi et al., 2004; Okutsu et al., 2007) and further employed in various other commercially important fish species, as well as endangered species (Lacerda et al., 2010; Higuchi et al., 2011; Morita et al., 2015; Yoshikawa et al., 2017; Lujić et al., 2018; Shang et al., 2018; Franěk et al., 2019, 2021) Additionally, the combination of germ cell *in vitro* culture, cryopreservation and transplantation is a powerful strategy for preserving precious genetic resources of endangered fish species (Iwasaki-Takahashi et al., 2020). In sturgeons, Pšenička et al., 2015 established a germ cell transplantation technique for Siberian sturgeon (*Acipenser baerii*) using sterlet (*Acipenser ruthenus*) as recipients and showed colonization of donor cells in the gonads of recipients. Ye et al., 2017 demonstrated that isolated germ cells from Chinese sturgeon (*Acipenser sinensis*) could colonize in Dabry’s sturgeon (*Acipenser dabryanus*) larvae. With the establishments of germ cell *in vitro* culture (Xie et al., 2019), cryopreservation (Pšenička et al., 2016; Ye et al., 2021) and gene editing techniques such as CRISPR/Cas (Baloch A. R. et al., 2019), surrogate reproduction technology is expected to be a powerful method to preserve and recover endangered sturgeon species. Moreover, surrogacy is also being applied in model species to investigate physiology of GSCs and recently it has been applied to improve gene editing procedures (Wong et al., 2011; Zhang F. et al., 2020, 2021). Germ stem cells have been proven to have the capability to incorporate into the recipient’s genital ridge, undergo gametogenesis and generate functional donor-derived gametes (Yoshizaki et al., 2012). In fishes, undifferentiated type A spermatogonia (A_und_), probably a small portion of the type A spermatogonia population, are considered as spermatogonial stem cells (SSCs). A_und_ retain the ability of self-renewal, differentiation, and transformation into different germ cell types (biopotency, De Rooij and Russell, 2000; Schulz et al., 2010). A_und_ are single cells and the largest germ cells in the fish testis, with a large nucleus (Xie et al., 2020). To obtain efficient germ cell *in vitro* culture, cryopreservation and transplantation, a highly purified population of A_und_ cells is essential. However, the proportion of A_und_ spermatogonia is very low in the gonads and further decreases with spermatogenesis, increasing the proportion of differentiated germ cells (Schulz et al., 2005). In past decades, density gradient centrifugation was commonly employed to enrich germ cells in several fish species (Rolland et al., 2009; Linhartová et al., 2014; Pšenička et al., 2016). Nevertheless, due to its low resolution capacity, density gradient centrifugation is not accurate enough to enrich high purity of homogenous cell populations with similar physiochemical properties, such as type A and type B spermatogonia. Flow cytometric cell sorting (FCM), as well as fluorescence-activated cell sorting (FACS), is a precise and efficient method to collect target cells according to cell size, shape, granularity, self-fluorescence properties and specific surface antibodies conjugated with florescence dyes. Mammalian SSCs have been sorted by FACS based on SSCs surface markers, such as α6-integrin, CD9, SSEA-4, GFRA1, THY1, and MCAM (Kokkinaki et al., 2011; Kanatsu-Shinohara et al., 2012; Valli et al., 2014). However, to date, fish SSCs surface markers, *sgsa-1*, as well as cytoplasm markers, such as *plzf*, *gfrα1*, and *nanos2*, have been identified in only a few species (Xie et al., 2020). Some commercially important or endangered species lack transgenic strains or specific molecular markers to label SSCs, presenting limitations in detecting and isolating SSCs. In past decades, type A and type B spermatogonia were enriched respectively from pvasa-GFP transgenic rainbow trout according to cell size and green fluorescent protein (GFP) intensity. Further studies have shown A_und_ efficient enrichment only based on light scatters properties in rainbow trout, Japanese charr (*Salvelinus leucomaenis*, Kise et al., 2012), Nibe croaker (*Nibea mitsukurii*) (Hayashi et al., 2014) and Pacific bluefin tuna (*Thunnus orientalis*) (Ichida et al., 2017). Therefore, light scattering properties are applicable in enriching type A spermatogonia without cell-labeling systems such as transgenes and cell surface antibodies which ease its application in a wide pallet of non-model species.

Sorted populations of testicular cells are expected to be valuable material to investigate gene expressions among different germ cell populations, ultimately enabling precise dissection of stem cell marker genes. The mechanisms regulating the differentiation of sturgeon germ cells have received a lot of interest. The current studies on sturgeon focus on gonadal transcriptome profiling between sexes or different gonadal development stages (Berbejillo et al., 2012; Hagihara et al., 2014; Yue et al., 2015; Fajkowska et al., 2016, 2019; Wang et al., 2017, 2019; Zhang X. et al., 2020). To date, the characteristics of distinct germ cell populations are not well understood in sturgeon. This has hindered the identification of GSCs marker genes. Investigation of whole tissue results in the measurement of gene expression levels that are averaged over a certain cell population. Therefore, FCM is an ideal tool for addressing gene expression profiles by producing various precisely purified germ cell populations.

In the present study, sterlet testes were utilized to establish a strategy of purifying sturgeon germ stem cells. We investigated the morphology of sterlet testes at different maturing stages. Then we isolated a type A spermatogonial population from different developing stages using FCM based on light scatter properties. Finally, in sorted germ cell populations, the expression profiles of key genes involved in germ cell development were analyzed and a presumptive A type spermatogonia marker was identified.

## Materials and Methods

This study was conducted at the Faculty of Fisheries and Protection of Waters (FFPW), University of South Bohemia in České Budějovice, Vodňany, Czech Republic. The facility has the competence to perform experiments on animals (Act no. 246/1992 Coll. ref. number 16OZ19179/2016–17214). Animal handling and experimentation were approved by the Ethics Committee on the Institutional Animal Care and Use Committee of the FFPW according to the law on the protection of animals against cruelty (reference number: MSMT-6406/119/2)

### Fish

Undifferentiated sterlet testes samples were collected from 4 to 6 month-old individuals (∼10 cm in total length and ∼5 g in weight), differentiated gonad samples were collected from 16 to 18 month-old individuals (∼52 cm in total length and ∼200 g in weight) and maturing gonad samples were collected from 2 year-old individuals (∼69 cm in total length and ∼632 g in weight). Fish were sacrificed by deep anesthesia in 0.1% 3-aminobenzoic acid ethyl ester methanesulfonate-222 (MS-222) (Sigma, St. Louis, MO, United States).

### Preparation of Testicular Cells

Dissociation of testicular cells was performed according to Xie et al (2019). Gonads of sterlet were washed in phosphate-buffered saline (PBS; Sigma-Aldrich, St Louis, MO, United States) containing antibiotics (50 μg/ml ampicillin, 200 U/ml penicillin, 20 μg/ml streptomycin; all from Sigma, pH 8.0) and minced into 1 mm^3^ pieces. Then pieces of tissue were dissociated by 0.25% trypsin (Gibco) in PBS for 2 h. The digestion was stopped by a L-15 medium with 20% fetal bovine serum (FBS), filtered through a 40 μm pore-size nylon screen and centrifuged at 300x*g*. The cell pellet was resuspended in PBS.

### Flow-Cytometric Cell Sorting of Testicular Cells

**FIGURE 1:**
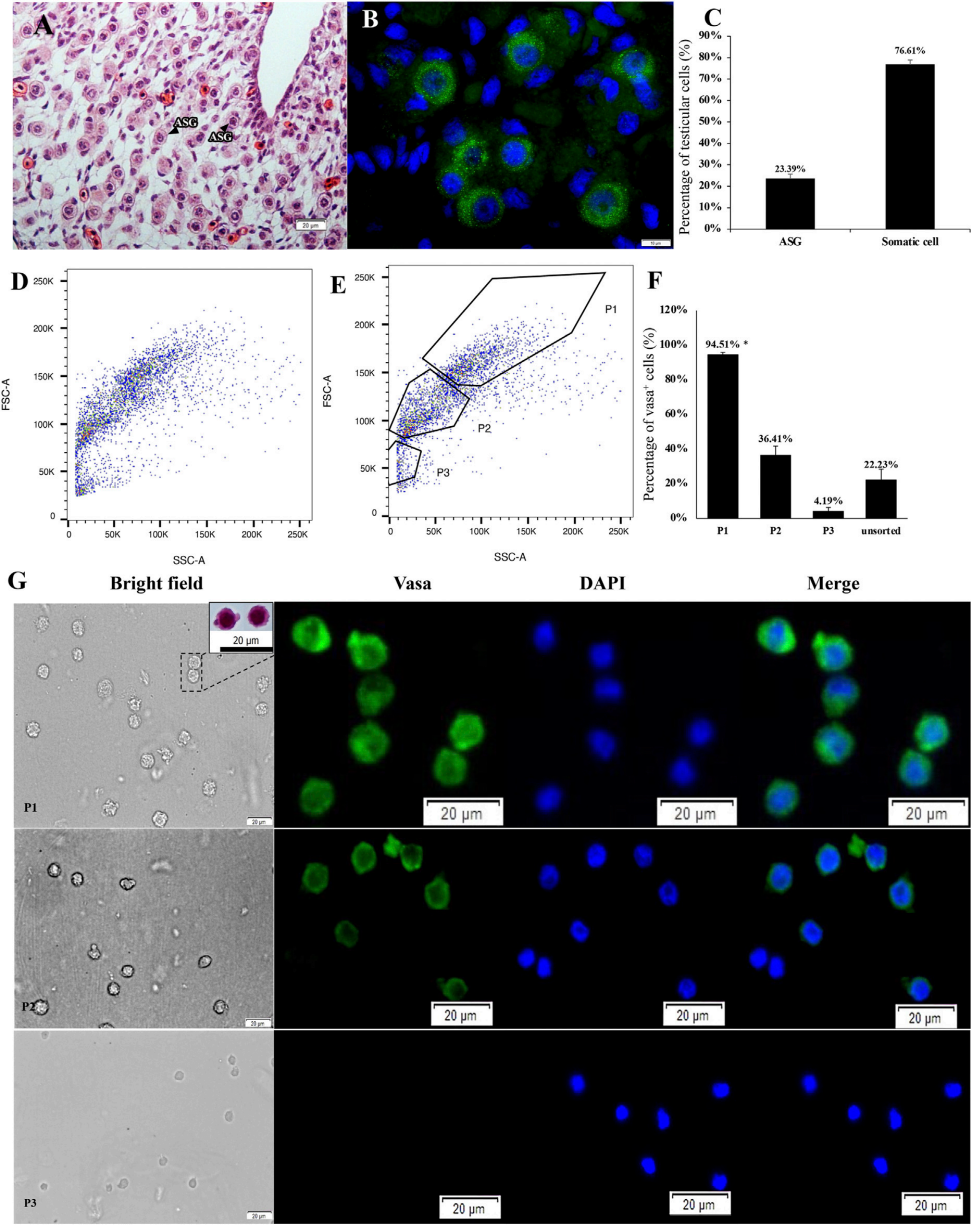
Histological observation and light scattering property-dependent fractionation of 6 month-old sterlet testes. **(A)** Testes section stained with hematoxylin and eosin (HE). **(B)** Testes section stained with anti-vasa antibody. **(C)** The proportion of germ cells and somatic cells in testes. ASG, type A spermatogonia. **(D, E)** Bivariate histograms of flow cytometry data from dissociated testicular cells prepared from 6 month-old sterlets. Gates P1-P3 were set on the histogram and used for cell sorting. **(F)** Vasa-positive rates of sorted cells in gates P1-P3 and unsorted live cells [mean ± standard deviation of mean (SD)]. Means with an asterisk indicate a significant difference (n = 6, *p* < 0.05). **(G)** Microscopic observation of sorted cells using gates P1-P3 and HE stained cells from gate P1 (small rectangle). Immunofluorescent staining for detection/calculation of vasa positive cells in different gates. Blue signal, DAPI staining, Green signal, vasa antibody staining.

**FIGURE 2:**
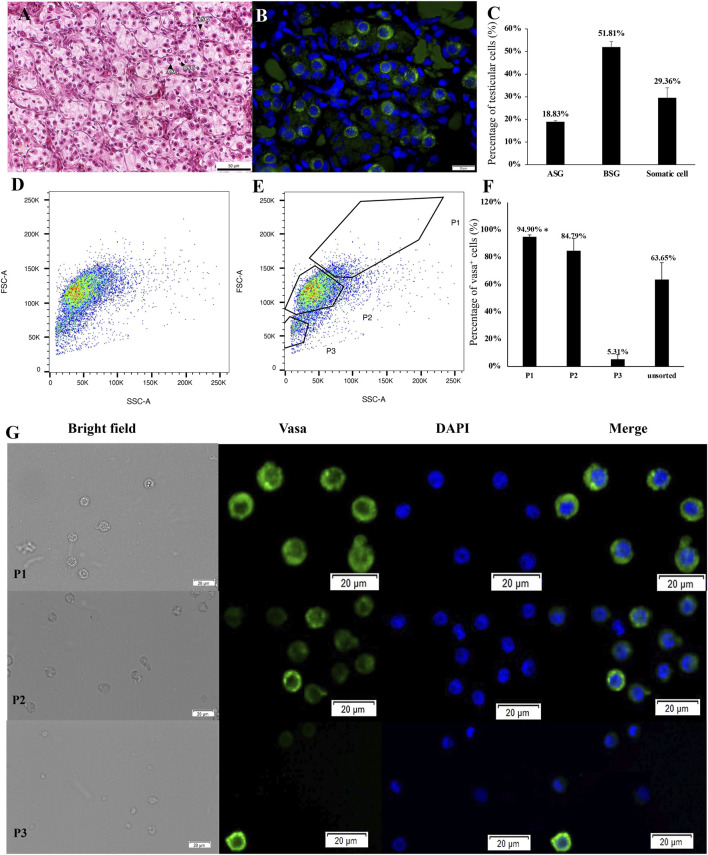
Histological observation and light scattering property-dependent fractionation of 18 month-old sterlet testes. **(A)** Testes sections stained with hematoxylin and eosin (HE). **(B)** Testes sections stained with anti-vasa antibody. **(C)** The proportion of various germ cell populations and somatic cells in testes. ASG, type A spermatogonia; BSG, type B spermatogonia. **(D, E)** Bivariate histograms of flow cytometry data from dissociated testicular cells prepared from 18 month-old sterlets. Gates P1-P3 were set on the histogram and used for cell sorting. **(F)** Vasa-positive rates of sorted cells in gates P1-P3 and unsorted live cells [mean ± standard deviation of mean (SD)]. Means with an asterisk indicate a significant difference (n = 6, *p* < 0.05). **(G)** Microscopic observation of sorted cells using gates P1-P3. **(H)** Cells from gates P1-P3 stained with anti-vasa antibody and DAPI.

**FIGURE 3:**
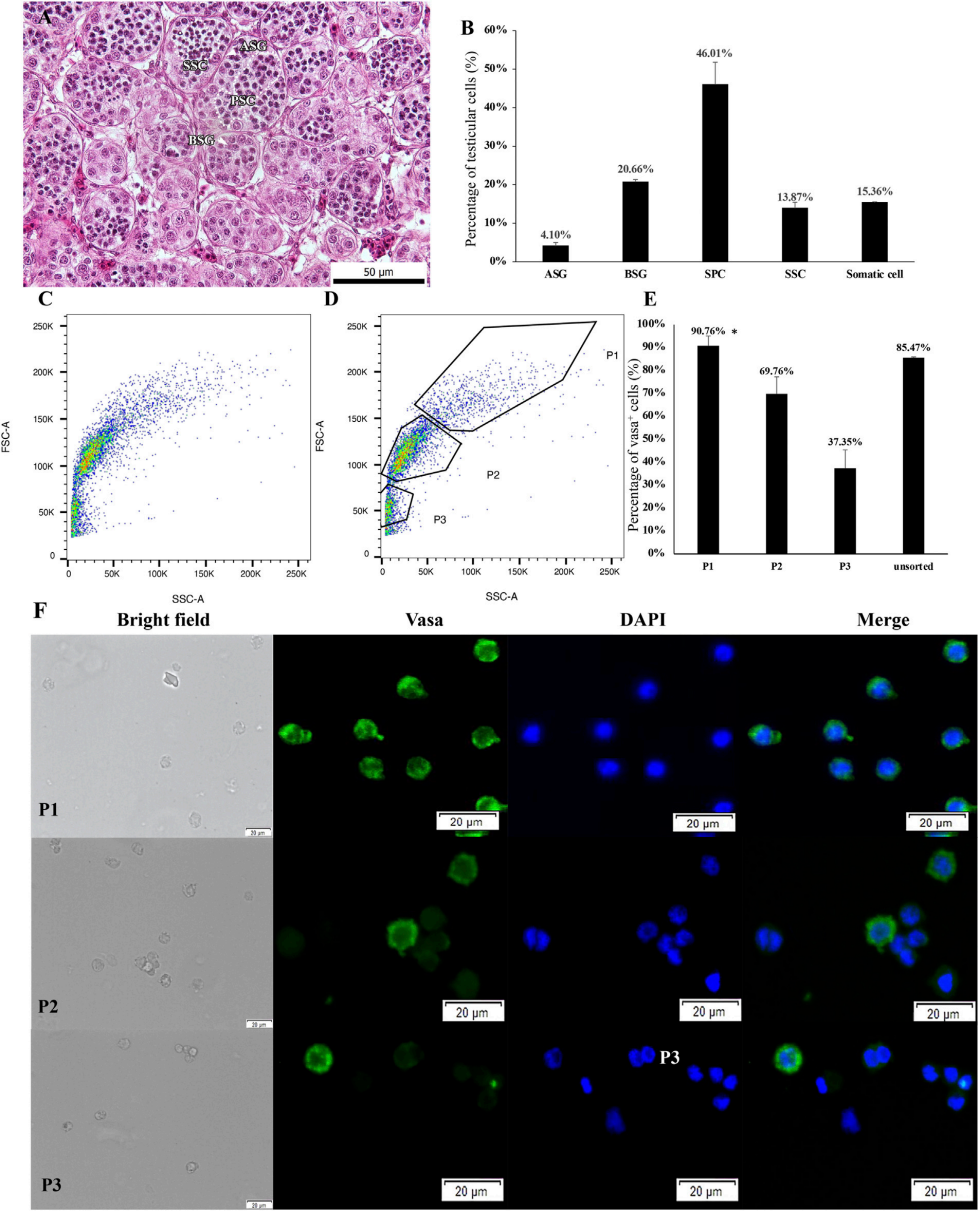
Histological observation and light scattering property-dependent fractionation of 2 year-old sterlet testes. **(A)** Testes sections stained with hematoxylin and eosin (HE). **(B)** The proportion of various germ cell populations and somatic cells in testes. ASG, type A spermatogonia; BSG, type B spermatogonia; SPC, primary spermatocyte; SSC, secondary spermatocyte. **(C, D)** Bivariate histograms of flow cytometry data from dissociated testicular cells prepared from 2 year-old sterlets. Gates P1–P3 were set on the histogram and used for cell sorting. **(E)** Vasa-positive rates of sorted cells in gates P1–P3 and unsorted live cells [mean ± standard error of mean (SEM)]. Means with an asterisk indicate a significant difference (n = 5, *p* < 0.05). **(F)** Microscopic observation of sorted cells using gates P1–P3. **(G)** Cells from gates P1-P3 stained with anti-vasa antibody and DAPI.

FCM of testicular single-cell suspensions was performed using a cell sorter - BD FACSAriaTM Fusion (BD Biosciences, United States). The cells were sorted using a standard sterile setup of the cell sorter: 100 µm nozzle, 20 psi, PBS as a sheath fluid and precooled down to +5°C sample holder. One hundred cells suspensions of testicular cells prepared as described in (2.2) were sorted into 384 well plates for q-PCR studies, and onto poly-L-lysine coated slides for further morphological observation and immunocytochemistry studies. The isolation of different cell populations was performed according to their forward and side scatter (FSC-A and SSC-A) values as demonstrated on flow cytometry plots of Figures 1–3. For the purpose of live/dead cells separation the viability dye (Hoechst 33342) was added to all of the samples in a final concentration of 1 μl/ml. Prior to gating the strategy debris, doublet discrimination and live/dead cells separation were performed. Dead cells were gated out according to their positive signal from the Hoechst 33342 staining (Supplementary Figures S1, S2).

### Immunolabeling

The sorted cells from the defined gates were mounted on microscope glass slides and fixed with 4% paraformaldehyde in PBS for 1 h. The fixed cells were washed in PBS and permeabilized with 0.3% TritonX-100. The slides were blocked in PBS with 1% BSA and 0.05% Tween 20 (blocking solution) for 40 min. Then the slides were incubated overnight at 4°C with DDX4 (vasa) rabbit polyclonal antibody (dilution 1:300, final concentration 1.8 μg/ml, GTX116575, GeneTex), which is specific for sturgeon germline cells (Pšenička et al., 2015), and exposed for 1 h at room temperature with secondary antibody anti-rabbit IgG–fluorescein isothiocyanate (FITC; F0382, Sigma, dilution 1:50) followed by staining with 4,6-diamidino-2- phenylindole solution (3 ng/ml). The slides were covered with a coverslip and observed under a fluorescence microscope IX83 (Olympus, Japan) equipped with an ORCA R2 camera (Hamamatsu Photonics, Japan), and processed using the CellSens Olympus software. For each sample, a minimum of 800 cells was counted to calculate the proportion of vasa-positive cells; the proportion was expressed as a percentage of the total living testicular cells.

The immunohistochemistry of gonads was modified according to Pšenička et al., 2015. Testes were serially sectioned by a CM1850 Cryostat according to the standard cryo-section method. Subsequent blocking and incubation were processed as described for the immunocytochemistry of sorted cells.

### Histology

Sterlet gonad samples as well as sorted cell samples were fixed with Bouin’s solution overnight, then dehydrated and embedded following the standard paraffin method. The paraffin block was sliced into 4.5 μm serial sections. The paraffin sections were stained with hematoxylin and eosin (HE). Different germ cell populations were identified and counted according to the description of Lacerda et al., 2014.

### RT-qPCR of Germ Cell Related Genes

A bulk of 100 cells was FCM in 5 μl of BSA solution and stored at −80 °C in a freezer. Reverse transcription was performed using 2 μl of sample with 3 μl of RNase-free water, 0.5 μl of oligo dT and random hexamers (50 μM each), 0.5 μl of dNTPs (10 mM each) and 0.5 μl of RNA spike (TATAA Universal RNA Spike, TATAA Biocenter). Samples were incubated for 5 min at 75°C, followed by 20 s at 25°C and cooled to 4°C. Then, 0.25 μl of SuperScript III Reverse Transcriptase (Invitrogen), 0.25 μl of recombinant ribonuclease inhibitor (RNaseOUT, Invitrogen), 0.5 μl of 0.1 M DTT (Invitrogen), and 2 μl of 5 × First strand synthesis buffer (Invitrogen) were added and incubated: 5 min at 25°C, 60 min at 50°C, 15 min at 55°C and 15 min at 75°C. The cDNAs obtained were diluted to a final volume of 80 μl and stored at −20°C.

The qPCR reaction contained 5 μl of TATAA SYBR Grand Master Mix, 0.5 μl of forward and reverse primers mix (mixture 1:1, 10 μM each), 2 μl of cDNA and 2.5 μl of RNase-free water in a final volume of 10 μl. The qPCR was performed using the CFX384 and CFX96 systems (BioRad) with the following conditions: initial denaturation at 95°C for 3 min, 40 repeats of denaturation at 95°C for 15 s, annealing at 60 °C for 20 s and elongation at 72°C for 20 s. Melting curve analysis was performed after to test reaction specificity and only one product was detected for all assays. All primers applied in this study were shown in Table 1.

**TABLE 1:** Primers for detection of mRNA in sorted cells.

Gene	Gene ID	Forward primer	Product length
		Reverse primer	
catenin beta-1 (β-catenine)	117400133	F: AGA​ACT​GAA​CCT​ATG​ACT​TGG​AAT​G	150
		R: AGC​TAG​GAT​CAT​CGT​GAC​GG	
deleted in azoospermia-like (dazl)	117435412	F: GTA​CAC​TGG​TGG​AGC​GCT​TA	130
		R:CTGTGGGGCAGCATACTGAT	
dead end (dnd)	117412941	F: GAG​TTC​CAG​TTG​ACA​CGC​TC	139
		R: TCC​ACT​CTC​GTC​CTG​TTT​TGT	
Boule	117413842	F: CTC​CCC​AGT​CAT​GGT​CAC​AC	130
		R:TCTACACGGGCTCCATACCT	
endothelin receptor Ba (ednrba)	30442	F: GGG​GAC​TTG​CTC​TAC​ATT​CTC	137
		R:CTGAGGACCGTGATTCCCAC	
E3 ubiquitin protein ligase (itch)	100331274	F: TTA​CGT​CCC​CTA​TCG​TAC​CCC	129
		R:AGGAGTGTTTGGGCAATGGT	
receptor tyrosine kinase (kitr)	30256	F: TTG​TCA​AAG​GCA​ATG​CAC​GG	141
		R:GGTAAGGGCTGCTGCCTAAA	
lymphocyte antigen 75 (LY75)	117406607	F: GAG​GAG​CAG​CGA​GTC​AAG​AT	117
		R: CAT​CGC​AGG​TTT​ACT​CGT​TTC	
probable ATP-dependent RNA helicase DDX4 (vasa)	117409552	F: AAG​AAC​ACA​ACT​CTT​ATT​GGA​GCA	148
		R: TGC​CAA​CTT​TCA​TTA​CAA​CAG​AAC	
zinc finger and BTB domain-containing protein 16-A (plzf)	117397484	F: TGA​AGC​CAG​ATC​ACA​GGA​GC	83
		R: GTGTTGTTCCACGGCGTC	

### *In situ* Hybridization (ISH)

RNAscope multiplex Fluorescent reagent kit v2 (cat.no. 323100) was used as instructed by Advanced Cell Diagnostics (ACD). A *plzf* probe was created by ACDbio as Ar-LOC117397484 targeting 102-1029bp of *Acipenser ruthenus* zinc finger and BTB domain-containing protein 16-A (Gene ID: 117397484). β-actin probe, named as Ar-actb1, targeting 102-1821bp of *Acipenser ruthenus* beta actin-1 (Gene ID: 117431529), was used as a positive control. A probe against the bacterial DapB gene (ACD) was used to as negative control. Samples were fixed in 10% neutral buffered formalin (NBF) for 20 h then dehydrated in an ethanol series followed by xylene. After cutting, tissue sections 5 μm thick were deparaffinized in xylene and 100% ethanol. Sections were then rehydrated with hydrogen peroxide, followed by antigen retrieval. After treatment with protease, hybridization with target probes, and amplification, labeling with Opal 520 (cat.no. FP1487001KT) was performed on tissues sections. The samples were mounted with fluoroshield 4′,6-diamidino-2-phenylindole (DAPI), covered with a coverslip, observed under a confocal microscope Olympus FV 3000, and processed with CellSens Olympus software.

A semi-quantitative scoring guideline according to the manufacturer’s instructions was applied to evaluate the staining results. The number of dots per cell was scored to correlate with the number of RNA copy numbers. The dynamic range of expression (cell-by-cell expression profiles) could be quantified for the entire tissue section or selected regions of interest by binning cells with different levels of expression into separate bins. Cells within each bin, characterized by number of dots, was estimated and calculated as follows:

**Table:** 

Score	Criteria
0	No staining or <1 dot/10 cells
1	1–3 dots/cell
2	4–9 dots/cell. None or very few dot clusters
3	10–15 dots/cell and <10% dots are in clusters
4	>15 dots/cell and >10% dots are in clusters

### Statistical Analysis

In 3.1–3.3, for the comparisons among different sorting gates, statistical significance was determined using a one-way ANOVA followed by Student-Newman-Keuls test using a statistical significance level of *p* < 0.05. All data are presented as the mean value ±standard deviation of the mean (SD). In 3.4, statistical analysis was composed of two comparisons; 1) P1 cells between undifferentiated *v*. differentiated testes and 2) P1 and P2 cells of differentiated testes. In both comparisons, we tested 10 genes (β-*catenine*, *boule*, *dazl*, *dnd1*, *ednrba*, *itch*, *kitr*, *LY75*, *plzf*, *vasa*) analyzed by qPCR, which were involved in gonad development of sturgeon and other fishes (Maouche et al., 2018; Vizziano-Cantonnet et al., 2018; Fajkowska et al., 2019, 2020; Wang et al., 2019; Xie et al., 2020). Cq values were normalized per group for each gene separately using either Cq values from the differentiated group in undifferentiated v. differentiated comparison or using P1 group in P1 v. P2 comparison with the formula:

$$Rq\;=2^{-\left(Cq-\;\bar{x}\right)}$$ (1)

Where Rq is relative quantification (value after normalization), Cq is the qPCR output value, $\bar{x}$ = arithmetic mean of Cq values from differentiated group or P1 according to the comparison. Note that this normalization is possible because all qPCRs were performed on 100 cells as input material. For statistical analysis, the Wilcoxon rank sum test was used. Since one comparison included 10 genes (*i.e*. 10 separate tests), the false discovery rate was controlled with the Benjamini–Hochberg procedure (Benjamini–Hochberg, 1995). The significance level was set as an adjusted *p*-value < 0.05. In 3.4, all data are presented as the geometric mean value ± geometric standard deviation (GSD). R statistical language (4.0.5) was used for all data analysis.

## Results

### Histological Observation and Flow-Cytometric Analysis of Undifferentiated Testes

According to the histological observations, in the 4 to 6 month-old testes of sterlet, spermatogonia were mostly large, single cells, surrounded by cytoplasmic extensions of one or two Sertoli cells (Figure 1A). Immunohistochemistry of vasa antibody indicated that the large, single cells were vasa-positive cells (Figure 1B). According to Ye (2014) and Pšenička et al., 2015, in sturgeon, vasa mainly expressed in germ cells but not in somatic cells. Therefore, the vasa-positive cells were expected to be type A spermatogonia, which were mostly in undifferentiated stage. Based on the histology observation of sturgeon gonadal development (Pšenička et al., 2015), the testes of sterlet were in maturity stage I: containing type A spermatogonia and somatic cells; 23.99 ± 2.63% of cells were type A spermatogonia (Figure 1C).

Cells stained with Hoechst 33342 and Propidium Iodide (PI) when analysed immediately after staining were showing double positive signal for dead cells and double negative signal for live cells (Supplementary Figure S2). A flow-cytometric sorting plot is demonstrated in Figure 1D. We set three gates to isolate type A spermatogonia (Figure 1E). Cells sorted from the P1 gate demonstrated high FSC-A and high SSC-A. In the P2 gate, FSC-A and SSC-A were lower than P1. Cells in P3 performed the lowest FSC-A and SSC-A. According to analysis of the immunocytochemistry, 94.51 ± 1.38% (mean ± SD) cells in the P1 gate were vasa positive, which is significantly higher than other gates and unsorted cells (n = 6, *p* = 0.01, Figures 1F,I). The vasa positive rate in P2 and P3 gates and unsorted living cells were 36.41 ± 5.43%, 4.19 ± 2.16% and 22.23 ± 6.23%. Cells in P1 were >10um in diameter, while cells from P3 gate were smaller. Cells in P1 also showed a round shape and a prominent nucleus (Figure 1G).

### Histological Observation and Fractionation of Differentiated Testes Using FCM

Germ cell cysts were observed in 18 month-old testes, containing 4–16 spermatogonial cells (Figure 2A). Compared with type A spermatogonia in 6 month-old testes, most spermatogonial cells were smaller, had one or more nucleoli and a relatively large cytoplasmic volume. They were also connected cells with a round to oval shape, indicating that type A spermatogonia have differentiated into type B spermatogonia in cysts. Type A and type B spermatogonia were tested as vasa-positive by immunohistochemistry (Figure 2B). The proportion of various germ cells is shown in Figure 2C. Thus, the testes of sterlet are in maturity stage II: containing type A and type B spermatogonia and somatic cells. The meiosis phase of spermatogenesis, however, did not occur.

The same gates set in 3.1 were applied to analysis FCM sorting plot of testicular cells from 18 month-old males (Figures 2D,E). As a result, cells collected in the P1 gate displayed 94.90 ± 1.42% vasa positive, which was significantly higher than P2 (n = 6, *p* = 0.036 < 0.05), P3 and unsorted cells (*p* = 0.01, Figure 2F). Cells from P1 also showed a similar diameter and large nucleus (Figures 2G,H). The total amount of cells located in the P2 gate increased, 84.79 ± 8.85% were detected as vasa-positive cells. Cell size was approximately 8–10 μm, relatively smaller than those cells sorted in the P1 gate (Figures 2G,H); 5.31 ± 3.33% cells showed vasa-positive signals in P3 (*p* = 0.001, Figure 2E).

### Histological Observation and Fractionation of Meiotic Phase Testes Using FCM

In 2 year-old testes, various germ cell populations were found, including type A, type B spermatogonia, spermatocytes and few developing spermatids (Figure 3A), indicating the testes developed into maturity stage III. The proportion of various of germ cells is shown in Figure 3B. The whole testicular cell suspension was sorted into the same setting used for 3.1 and 3.2 (Figures 3C,D). As a result, the rate of vasa-positive cells in P1 was 90.76 ± 4.24%, which is significantly higher than other gates (n = 5, *p =* 0.01, Figure 3E), except unsorted cells (*p =* 0.516). Cells located in the P1 area were the largest cells compared with cells in P2 and P3 (Figures 3F,G). The rates of P2, P3 and unsorted living cells were 69.76 ± 7.43%, 37.35 ± 8.02% and 85.47 ± 0.41% respectively (Figure 3E).

Among testes in stage I, II and III, cells sorted from P1 demonstrated similar morphological characteristics, such as a large cell size and high nucleocytoplasm ratio, and showed a high rate of vasa-positive cells (>90%). Thus, it could be deduced that the population sorted from the P1 gate contained mainly type A spermatogonia.

### Expression Profiles of Germ Cell Related Genes of Sorted Cells

To investigate the comprehensive phenotypic characterization of type A spermatogonia, the preferential expression of sorted cells was analyzed by microarray analysis. The expressions of 10 germ cell related genes were analyzed by RT-qPCR. To examine the germ cell characteristics from testes among different developmental stages, qPCR was performed on 100 cells enriched in the P1 fraction from undifferentiated and differentiated testes (stage I and II), respectively.

Expression of 10 genes in the P1 cells was detected at both developmental stages. Results showed that nine genes showed no significantly different expressions in P1 cells, which respectively sorted from testes at stage I and II (n = 3, *p* < 0.05). This indicated that regardless of developmental stages, germ cells sorted from gate P1 retain stable characteristics. The gene expressions of P1 and P2 gates from stage II testes were quantified. As a result, *dnd1*, *plzf*, *boule* and *kitr* were significantly down-regulated in P2 cells compared with those from P1 (n = 3, *p* < 0.05). Overall, considered with evidence from cell morphology and immunocytochemistry, our deduction was confirmed that the P1 gate collected high purity type A spermatogonia from testes of different maturation. What is more, at stage II testes, the P2 gate mainly sorted type B spermatogonia. Genes *dnd1*, *plzf*, *boule* and *kitr* showed high expressions in type A spermatogonia.

**FIGURE 4:**
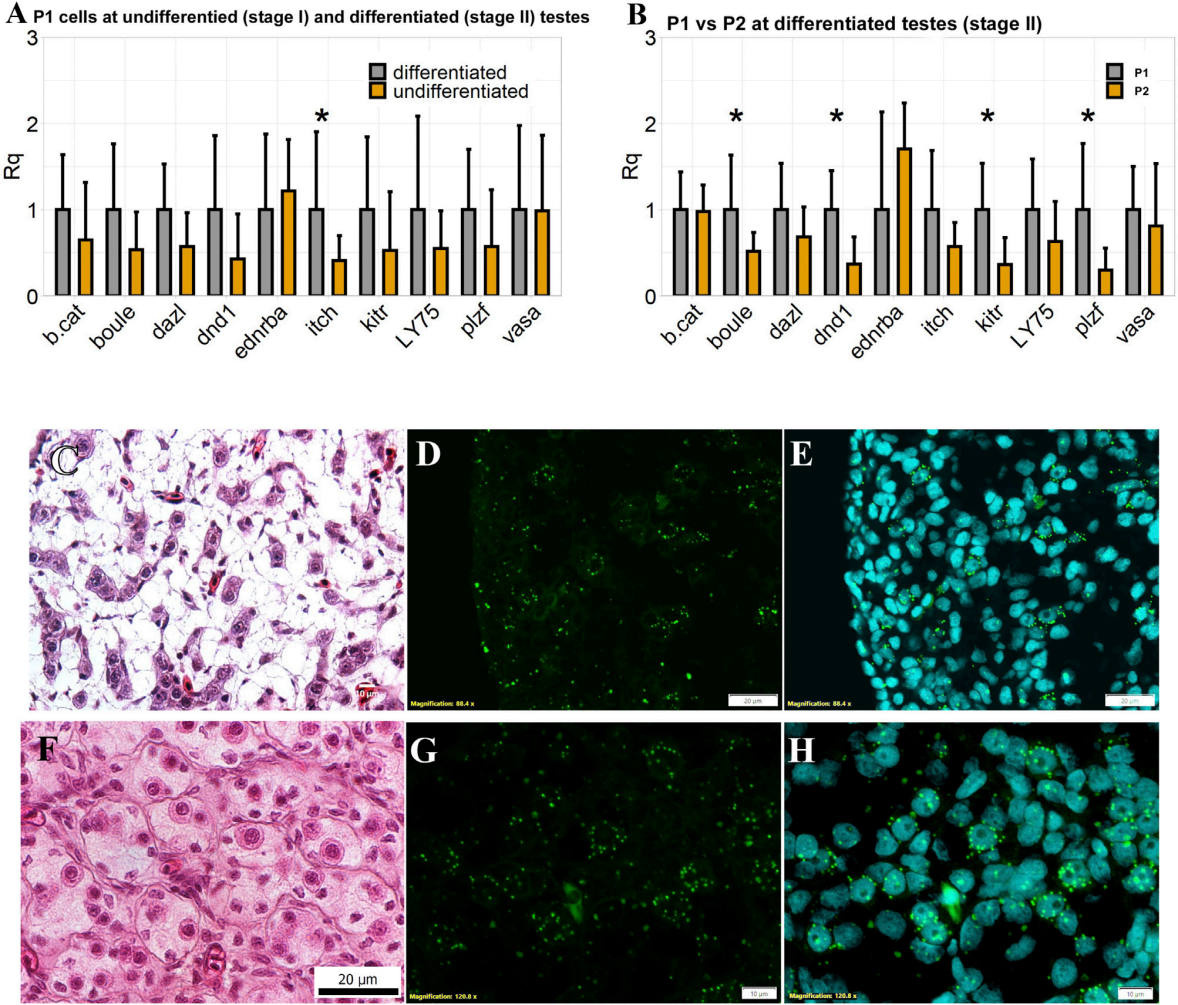
Expression of candidate markers for sorted cell populations. **(A)** Comparision of germ cell related genes expression profile of P1 cells between stage I and II testes by q-PCR. Gene expressions in stage I were defined as the relative value 1.0. **(B)** Comparision of germ cell related gene expression profiles of P1 and P2 cells at stage II testes by q-PCR. Gene expressions in P1 were defined as the relative value 1.0. Cq values were normalized per gene for relative quantification using the arithmetic mean of differentiated group values **(A)** and P1 values **(B)**. $Rq\;=2^{-\left(Cq-\;x^{3}\right)}$ . Data are presented as geometric mean value ± geometric standard deviation (GSD). Means with an asterisk indicate a significant difference (n = 3, *p* < 0.05). **(C)** Histological observation of early stage testes used for *in situ* hybridization (ISH). **(D)** The cellular distributions of *plzf* by ISH. **(E)** Merged photograph with DAPI. **(F)** Histological observation of testes at a later maturing stage used for ISH. **(G)** The cellular distributions of *plzf* by ISH. **(H)** Merged photograph with DAPI.

The cellular distributions of *plzf* in sterlet testes were analyzed by ISH assays. As it described in 2.7, number of distinctive dot is corresponded to the RNA copy number successfully bound with the probes. In Figure 4C, the testis tissue examined contained mostly type A spermatogonia. As shown in Figures 4D,E, the signal of *plzf* was found in type A spermatogonia, which were large single cells with a large nucleus and a prominent nucleolus. Ninety three percent of labeled cells were ranked as score 3, each cell had 10–15 dots. In addition, ISH was also performed in testes at a later maturing stage (Figure 4F). Eight to 32 cells could be observed in one germ cell cyst, indicating the testes contain type A and type B spermatogonia. As it shown also in Figures 4G,H, in one germ cell cyst, one large cell with a clear nucleolus was scored as 4 (>15 dots) or as 3 (10–15 dots), and 2–3 cells could be labeled as score 2 (4–9 dots). There were 67.00 ± 2.64% of cells in cysts without any detectable signals. It indicated that peak expression of *plzf* was in A_und_ and signals decreased in A_diff_, while it did not show evidence of labeling in type B spermatogonia. Therefore, *plzf* represents a potential marker to identify type A spermatogonia. Sorting gates properties and cell types in three sorting gates were summarized in Table 2.

**TABLE 2:** Summary of cell characteristics among sorting gates using flow cytometry.

		Gate		
		P1	P2	P3
Gate properties		High FSC-A	Medium FSC-A	Low FSC-A
		High SSC-A	Low SSC-A	Low SSC-A
cell types	stage I	ASG 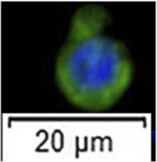	few ASG 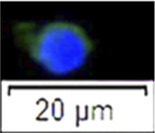	SC 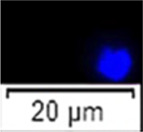
	stage II		BSG 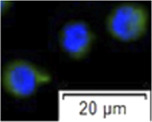	SC 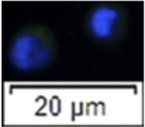
	stage III		BSG, spermatocyte 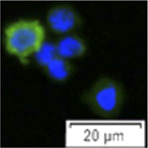	spermatocyte, SC 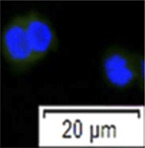
expressed marker genes		*plzf*, *kitr*, *dnd1*, *boule*	-	-

FSC-A, Forward scatter-Area; SSC-A,Side scatter-Area; ASG, type A spermatogonia; BSG, type B spermatogonia; SC, somatic cells.

## Discussion

FCM is a common technique to isolate specific cells in model species. However, in aquaculture and endangered species, such as sturgeon, transgenic strains are not available, as well as particular molecular markers identifying target cell lineages. In case of applying surrogate reproduction to endangered species, FCM using light-scattering properties can be a particularly useful tool to enrich type A spermatogonia. Moreover, the sorted cells can be utilized for downstream analysis (expression profiles and sequencing) as well for transplantation into recipients (Ichida et al., 2019).

In the present study, we established a method using FCM to enrich type A spermatogonia from sterlet testes at different maturation stages, and reported phenotypic characterization of different germ cell populations at different developmental stages. Our strategy indicated that a highly pure type A spermatogonia population could be reliably sorted by flow-cytometry with high forward scatter and high side scatter parameter values from testes at different stages of maturation. Sorted cells were available to undertake high-resolution molecular analysis.

According to the histological observation of fish testes, type A spermatogonia are the largest cells among all the germ cells throughout the testes developmental stage and possess the ability of “stemness” (Xie et al., 2020). Our results demonstrated that cells sorted from the P1 gate (both high FSC-A and SSC-A values) showed very high vasa positive rates at maturity stage I. The morphological characteristics of sorted cells, such as size and the nucleocytoplasmic ratio, were identical to type A spermatogonia by histological observations. In stage II and III, vasa-positive cells collected from P1 showed similar characteristics as those from stage I testes. It indicated that in all three stages, vasa-positive cells collected from the P1 gate might be type A spermatogonia. To confirm this sorting result, we collected cells from P1, P2 and P3 and detected the germ cell related gene expressions. With the comparison of gene expressions in P1 cells between undifferentiated and differentiated testes (stage I and II), most germ cell related genes had similar expressions, indicating cells in P1 represented the same cell population. Therefore, the present sorting strategy allowed us to purify type A spermatogonia at different developmental stages.

At stage II, testes are differentiated. Compared with undifferentiated testes, the sorting plot based on a light scattering property has obviously been changed. More cells located in P2 gate, showing relatively low FSC-A and SSC-A, suggesting these cells are smaller than cells in P1 and have a simple intracellular structure. The vasa-positive rate of cells in P2 was higher than the cells in P2 stage I. The histology of testes revealed that type B spermatogonia have increased in this stage. Thus, the difference of the sorting plot may have been caused by a change in the proportion of germ cell populations. Vasa-positive cells in the P2 gate are expected to contain type B spermatogonia. Furthermore, we compared expressions of germline related genes of P1 and P2 cells at stage II. Most genes were more down-regulated in P2 cells than in P1. Expressions of *dnd1*, *plzf, boule* and *kitr* were significantly decreased in P2 cells. Combined with results of the FCM, these differences may be caused by increasing numbers of type B spermatogonia.

The other interesting phenomenon we observed in this study was that the side scatter value of the whole testicular cells increased along with the growing forward scatter. The factors affecting total light scatter include the membrane, nucleus, internal complexity of the cell, cell shape and surface topography. Type A spermatogonia of Pacific bluefin tuna were enriched in the fraction with high FSC-A and relatively low SSC-A values (Ichida et al., 2017). This was also shown in Nibe croaker, rainbow trout and masu salmon (Kise et al., 2012), indicating type A spermatogonia possess largest size and a relatively simple internal cell structure in these species. It assumed that the light scattering properties of type A spermatogonia might be widely conserved throughout the evolution of various teleosts. However, in the present study, type A spermatogonia in the high FSC-A fraction always showed high SSC-A, especially in undifferentiated testes; most germ cell are undifferentiated spermatogonia. Studies of intracellular ultrastructure of sturgeon germ cells, however, are needed in the future. Similar sorting plots have been reported in medaka (*Oryzias latipes*) testicular cells. Spermatogonia at early stages were enriched in a fraction with both a high FSC-A and SSC-A signal (Satoh et al., 2019). Monitoring light scatter properties of human pluripotent stem cells, Ramirez et al. (2013) revealed that high SSC-A cells were characterized by more frequent simultaneous expression of the cell surface pluripotency factors and displayed a higher mitochondrial content. High SSC-A cells were more likely to generate colonies upon single-cell passage than low SSC-A cells. Therefore, it will be of interest to determine whether the side scatter intensity also reflects the potential of differentiation in fish germ stem cells.

In the present study, nine of 10 genes showed no differential expressions in P1 cells from both stages I and II. By comparing the cell morphology and composition of cell types in the testes, we speculate that P1 mainly contains the same cell population. Interestingly, although most genes showed no differences, gene expressions of P1 cells at stage I were generally lower than those at stage I. In addition, expressions of most genes were down-regulated from P1 to P2 cells at stage II. *dnd, boule, plzf* and *kitr* showed significant reduction in P2 cells. Only *ednrba* showed higher expression in P2 than that in P1 cells. What is more, the apparent cellular distributions of *plzf* were detected in the cytoplasm of type A spermatogonia in sterlet testes by *in situ* hybridization. It is worth noting that, in early stage testes, *plzf* showed lower expression (10–15 dots) in type A spermatogonia, while higher expression (>15 dots) was observed in type A spermatogonia at later maturing stages, which is consistent with the expression according to q-PCR. This heterogeneous expression is probably attributable to a different temporal stage of a functionally homogenous cellular population (Schulz et al., 2010; Santos Nassif Lacerda et al., 2013). In early developmental testes, high levels of transcription may be not fully activated. This finding was also observed in 5–6 month-old Siberian sturgeon gonads. Transcriptomic analysis revealed that 37 genes were activated in the ovary while only one gene was up-regulated in the testes (Vizziano-Cantonnet et al., 2018). Low expressions of germ cell related genes were also detected in 9 month-old Russian sturgeons (*Acipenser gueldenstaedtii*; Hagihara et al., 2014), suggesting that testes development has not yet been initiated. Therefore, *plzf* is expected to be an available marker of type A spermatogonia in sturgeon. To date, *plzf* is a transcriptional repressor essential for the maintenance of SSCs in mammals (Buaas et al., 2004). *plzf* was also demonstrated in SSCs of fishes, such as the zebrafish (*Danio rerio*) (Ozaki et al., 2011; Mohapatra and Barman, 2014), rainbow trout (Bellaiche et al., 2014), rohu (*Labeo rohita*) (Panda et al., 2011), dogfish (*Scyliorhinus canicular*) (Gautier et al., 2014), and several species of catfishes (Shang et al., 2015; Nayak et al., 2016; Lacerda et al., 2019). In the present study, *plzf* was first time identified as a type A spermatogonia marker in sturgeons.

*dnd* is a highly specific gene expressed in germ cells which plays an essential role in germ cell development. *dnd* was first isolated and identified in zebrafish where it was specifically expressed in germline cells (Weidinger et al., 2003). As one of the unique components of vertebrate germplasm, *dnd* is essential for the development and gametogenesis of primordial germ cells (PGC). Subsequently, *dnd* has been isolated in species such as mice (*Mus musculus*), African clawed toad (*Xenopus laevis*), chicken (*Gallus gallus*), medaka, and Atlantic salmon (*Salmo salar*) (Youngren et al., 2005; Horvay et al., 2006; Aramaki et al., 2008; Liu et al., 2009; Nagasawa et al., 2013). In sturgeon, *dnd* expression was abundant in spermatogonia and gradually became reduced in the late spermatogenic stages (Linhartová et al., 2015; Yang et al., 2015). In the present study, *dnd* demonstrated a decreasing expression between cells in P1 and P2 gates. In the testis, the expression of *boule* was found in meiotic germ cells in a fruit fly (*Drosophila melanogaster*) (Eberhart et al., 1996), mouse and human (Xu et al., 2001). Meiotic-preferential expression was noted in medaka (Xu et al., 2009) and rainbow trout (Liu et al., 2009). In Chinese sturgeon, expression of *boule* showed a peak in spermatogonia, then declined in primary and secondary spermatocytes and was faint in spermatids (Ye et al., 2015). In the present study, higher expression of *boule* was detected in cells sorted from P1 than P2 at stage II. *kitr* was also expressed more significantly in P1 cells than P2. Thus, we speculate *kitr* is also a potential marker of type A spermatogonia in sturgeon. The *Kit*/*kitlg* pathway can act on germ stem cells before spermatogenesis. Studies reported that mouse expression of *kitr* and *kitlg* was observed in the embryonic gonads prior to spermatogenesis onset. *kitr* and *kitlg* support proliferation and survival of PGC by reducing apoptosis of germ stem cells (Dolci et al., 1991; Runyan et al., 2006). In zebrafish, proliferation of PGC was promoted by a feeder layer expressing *kitlga*. In rainbow trout, *kitlgb* was found to stimulate autocrine in male germ stem cells before spermatogenesis (Maouche et al., 2018). The expression of *kitr* in sterlet, a species of sturgeon, spermatogonial stem cells has been observed for the first time in the present study. Further investigations will be required to determine how *kitr* accomplish this function in germ stem cells in sturgeon.

Given these findings, we considered that at stage II, cells sorted from the P1 gate were different from those from P2. Cells in P1 was supposed to include mostly type A while P2 enriched type B spematogonia. *dnd, boule, plzf* and *kitr* performed highest expression in a sturgeon spermatogonial stem cell during germ cell differentiation.

In the present study, we established an efficient method using FCM based on light scatter properties to enrich sterlet type A spermatogonia. Our method can stably purify type A spermatogonia among all pre-spermiogenic stages. Considering the yield and percentage of type A spermatogonia among testicular development, it will be more efficient to harvest type A spermatogonia from 16 to 20 month old sterlet. To our knowledge, this is the first demonstration of sturgeon germ cell sorting using the FCM platform, which combines cell enrichment with cluster isolation and characterization of type A spermatogonial populations. It might not only help to increase transplantation efficiency, but also give some insight into germ cell differentiation in sturgeons. In future studies, it will be of interest to utilize the purified type A spermatogonial population as an ideal material at the transcriptomic or proteomic analyses and accelerate various studies regarding the basic and applied biology of fish spermatogonia. Future research could also seek to extend this work to other germ cell populations, and accommodate it to other endangered fish species.

## Data Availability

The original contributions presented in the study are included in the article/Supplementary Material, further inquiries can be directed to the corresponding author.

## Supplementary Material

Supplementary Figure S1 Flow cytometry gating strategy. **(A)** Debris is gated out according to the small light scatter parameters on the bivariate plot of the Forward Scatter (FSC-A) and Side Scatter (SSC-A), **(B)** Doublet events are gated out according to the correlation of Area and Hight of the Side Scatter parameter signal, **(C)** Dead cells are gated out according to their positive signal after Hoechst 33342 staining (final concentration 1 µl/ml).

Supplementary Figure S2 Microscopy images showing sterlet testicular cells double stained with PI and Hoechst 33342 for live/dead separation. **(A, B)** images are showing cells positive for fluorescent signal from Propidium Iodide (PI) and Hoechst3342 staining (white arrows), which are the dead cells (apoptotic cell pointed at with an white arrow in image **(B)**. In the merge images we can also see the live cells (red arrows) that are showing the morphology of live cells with intact membranes and negative fluorescent signal for PI and Hoechst 33342 staining.
