# Supplementary figures and images for: Isolation and Characterization of Highly Pure Type A Spermatogonia From Sterlet (Acipenser ruthenus) Using Flow-Cytometric Cell Sorting
Source: Front Cell Dev Biol. 2021 Dec 10;9:772625. doi: 10.3389/fcell.2021.772625 (PMC8708567; doi:10.3389/fcell.2021.772625)

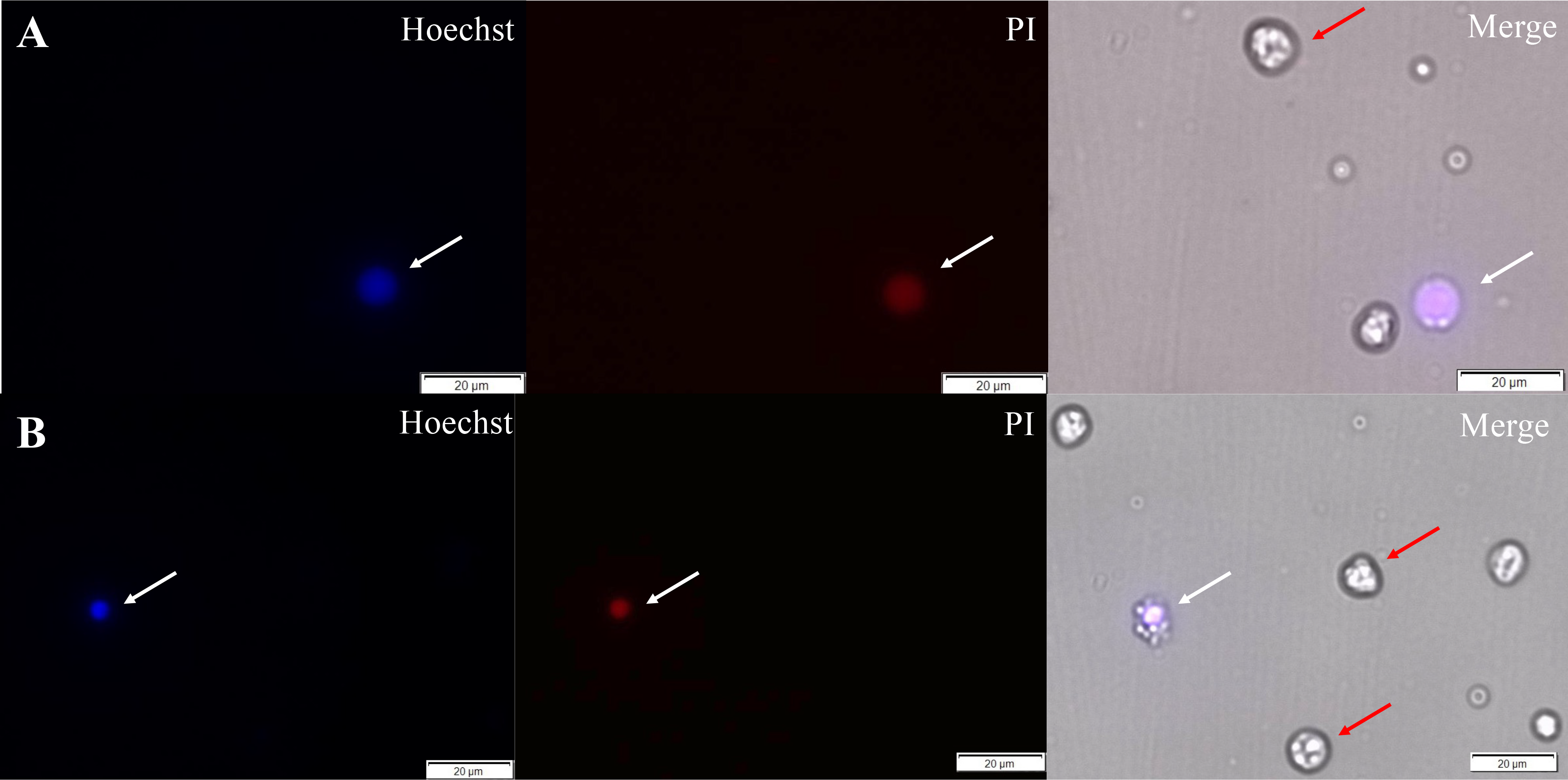

Supplement: Supplementary file 1 [file Image2.TIF]

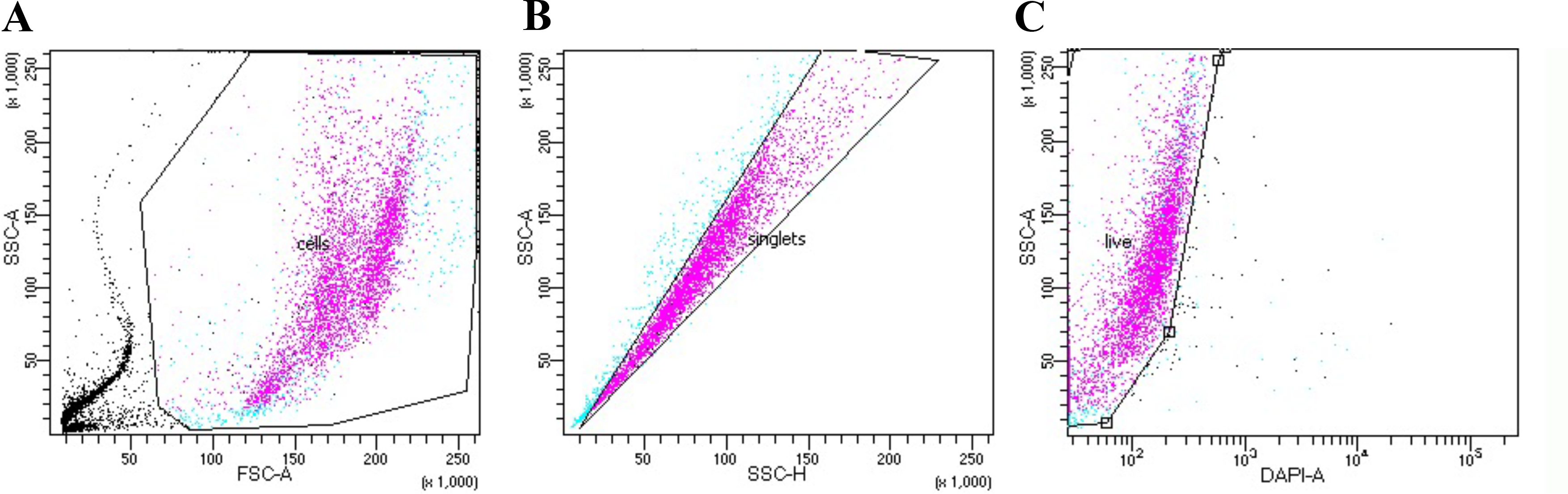

Supplement: Supplementary file 2 [file Image1.TIF]
